# Automated Peak Annotation in Time-of-Flight Secondary Ion Mass Spectrometry via a Physics-Informed Probabilistic Framework

**DOI:** 10.3390/molecules31132388

**Published:** 2026-07-07

**Authors:** Jiahua Chen, Yujie Cao, Xingyu Jiang, Chunpeng Wu, Qing Hao, Yun Hu, Jiahui Liu

**Affiliations:** Suzhou National Laboratory, Suzhou 215006, China; chenjh01@szlab.ac.cn (J.C.); caoyj@szlab.ac.cn (Y.C.); jiangxy@szlab.ac.cn (X.J.); wucp@szlab.ac.cn (C.W.); haoq@szlab.ac.cn (Q.H.); huy@szlab.ac.cn (Y.H.)

**Keywords:** ToF-SIMS, peak annotation, factor graph, belief propagation, probabilistic inference, physics-informed constraints, secondary ion mass spectrometry

## Abstract

Peak annotation in Time-of-Flight Secondary Ion Mass Spectrometry (ToF-SIMS) is a persistent bottleneck that typically requires the manual assignment of chemical formulas to hundreds of fragment ion peaks per spectrum. This work describes a physics-informed probabilistic framework that automates this task by combining five chemically motivated constraints—Gaussian mass accuracy, element composition priors, isotope pattern matching, nitrogen rule parity, and graded valence bounds—into a multiplicative belief score. We evaluate the framework on 643 ground-truth peaks from 151 compounds spanning both positive and negative ion modes, and we explicitly distinguish two regimes. As a scoring task—when the correct formula is present in the candidate list—the framework attains 52.3% Top-1 and 76.4% Top-3 accuracy, a 4.9-fold improvement over mass-only scoring. In fully automated end-to-end deployment, where candidates are generated de novo, Top-1 accuracy is 26.3%; the limiting factor is candidate generation rather than scoring, as only 46.5% of ground-truth formulas are currently produced by the database and combinatorial generator. Leave-One-Compound-Out Cross-Validation (59 compounds, 525 peaks) yields 51.8% Top-1 accuracy with fixed domain-knowledge weights, confirming generalization stability. Ablation analysis identifies element composition priors as the dominant non-mass constraint (−27.7 percentage points when removed), followed by isotope matching (−10.3 pp) and the nitrogen rule (−5.3 pp). The framework requires no labeled training spectra—relying instead on physically motivated priors and curated fragment databases—provides interpretable per-constraint scores (which represent relative rankings rather than calibrated probabilities), and supports polarity-specific configurations, offering a practical computational foundation for automated ToF-SIMS spectrum interpretation.

## 1. Introduction

### 1.1. Time-of-Flight Secondary Ion Mass Spectrometry

Time-of-Flight Secondary Ion Mass Spectrometry (ToF-SIMS) operates by bombarding a solid sample surface with a focused primary ion beam, ejecting secondary ions that are mass-separated according to their flight time [1,2]. The technique combines extreme surface sensitivity—sampling depths of 1–2 nm—with molecular specificity through fragment ion detection and can map chemical distributions with sub-micrometer spatial resolution in imaging mode. Current commercial instruments acquire spectra in two principal modes: “data1” (bunched) mode, which delivers high mass resolution (m/Δm>5000) at moderate spatial resolution, and “data2” (burst-aligned) mode, which prioritizes spatial resolution at the cost of reduced mass precision [3,4].

A single ToF-SIMS spectrum typically contains hundreds to thousands of peaks between 1 and 1000 Da, predominantly singly charged (z=1) positive or negative ions. The keV energy primary ion impact generates rich fragmentation patterns unlike those encountered in softer ionization techniques. Whereas electrospray ionization mass spectrometry (ESI-MS) produces predominantly intact molecular ions with controlled fragmentation, ToF-SIMS yields surface-specific fragments whose identities depend heavily on matrix effects, primary ion species, and sample preparation conditions [5].

### 1.2. The Peak Annotation Challenge

Assigning chemical formulas to observed peaks is the central interpretive task in ToF-SIMS data analysis, and it remains surprisingly difficult even for experienced analysts. Consider m/z 28.006: the nominal mass 28 could correspond to $N_{2}^{+}$ (28.006 Da), ${\mathrm{CO}}^{+}$ (27.995 Da), $C_{2}H_{4}^{+}$ (28.031 Da), or ${\mathrm{Si}}^{+}$ (27.977 Da). The ambiguity only worsens at higher masses, where the candidate space grows combinatorially. At m/z 69, for instance, ${\mathrm{CF}}_{3}$ (68.9952 Da) [2] and $C_{3}{\mathrm{HO}}_{2}$ (68.9976 Da) differ by a mere 0.0024 Da—well below what most ToF-SIMS instruments can resolve (50–200 ppm, corresponding to ∼0.003–0.014 Da in this mass range) [6].

Three compounding factors exacerbate this problem in ToF-SIMS specifically:

*(a) Near-isobaric formula ambiguity.* The limited mass accuracy of ToF-SIMS (typically 20–100 ppm, compared to <1 ppm for Orbitrap instruments) means that many distinct chemical formulas fall within the same mass tolerance window. In the 50–300 Da range, each m/z bin typically harbors 5–20 plausible candidates [7].

*(b) Matrix-effect-distorted isotope patterns.* Isotope pattern matching, a workhorse disambiguation tool in high-resolution MS, loses much of its power in ToF-SIMS. Strong matrix effects distort relative isotope intensities [8], and the detector response varies non-linearly with local ion concentration, unpredictably suppressing or enhancing isotope peaks.

*(c) Scarcity of annotated training data.* Unlike ESI-MS, where large spectral libraries (NIST, MassBank, mzCloud) exist, ToF-SIMS has no comprehensive annotated dataset, ruling out supervised machine learning approaches [9]. Ground-truth assignment still demands expert knowledge of surface chemistry and fragment ion physics.

In current practice, analysts annotate spectra manually using commercial software (e.g., IONTOF SurfaceLab) that offers library lookup but no automated probabilistic ranking of competing candidates [4,10]. This workflow is slow and operator-dependent and does not scale to the thousands of spectra generated in imaging experiments.

### 1.3. Existing Approaches and Their Limitations

A number of strategies have been brought to bear on mass spectral peak annotation, but each faces shortcomings when applied to ToF-SIMS:

*Rule-based sequential filtering*. Classical approaches apply hard constraints sequentially—mass accuracy first, then double bond equivalence (DBE), the nitrogen rule, and element ratio bounds [9,11]. Although effective at eliminating impossible candidates, sequential filtering cannot rank the remaining plausible formulas and provides no probabilistic confidence estimates.

*Database lookup tools*. Commercial ToF-SIMS software packages offer library search functionality that matches observed peaks against known compound databases [10,12]. These tools rely on exact or near-exact mass matches and lack any mechanism for resolving ambiguities when multiple database entries fall within tolerance. Curated reference collections such as the SurfaceSpectra Static SIMS Library [13], which compiles interpreted positive and negative ion spectra for more than 1000 materials, are invaluable aids for manual assignment but likewise offer no automated probabilistic ranking when several entries fall within mass tolerance.

*High-resolution MS tools*. Software such as SIRIUS [14] and MS-FINDER [15] performs sophisticated formula identification for LC-MS and GC-MS data. However, these tools exploit mass accuracies of <5 ppm and well-characterized MS/MS fragmentation trees—information that is simply not available in ToF-SIMS. The much larger mass uncertainty (typically 50–200 ppm) and the absence of tandem MS capability make these tools a poor fit.

What appears to be missing from the literature is a method that provides joint probabilistic inference combining multiple physical constraints specifically for ToF-SIMS peak annotation. The key unmet needs are: (a) a principled probabilistic framework that combines constraints multiplicatively rather than sequentially, (b) calibrated uncertainty estimates, and (c) a mechanism for inter-peak consistency reasoning.

### 1.4. Contributions

**Figure 1:**
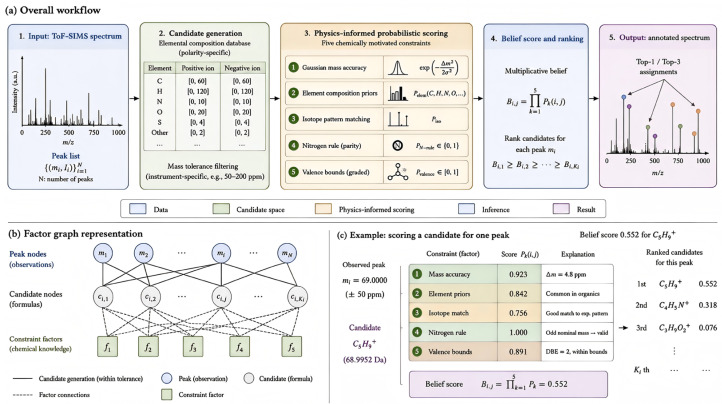
Overview of annotation framework. (**a**) Pipeline schematic: raw spectrum → peak detection → candidate generation (database lookup + DFG) → constraint scoring → ranked output. (**b**) Factor graph representation: variable nodes (circles, one per peak) connect to factor nodes (squares, one per constraint). (**c**) Score aggregation workflow: raw scores → batch normalization (min-max to [0.1, 1.0]) → power weighting → multiplicative belief.

This paper makes the following contributions. First, we cast ToF-SIMS peak annotation as probabilistic inference on a factor graph, where variable nodes represent peak-to-formula assignments, and factor nodes encode physical/chemical constraints. Second, we introduce five constraint functions, each derived from chemical physics: mass accuracy (Gaussian likelihood), element composition prior (log-prior over element frequencies), isotope pattern matching (theoretical distribution comparison), nitrogen rule (parity check), and valence reasonableness (graded H/C and O/C bounds). Third, we show that a computationally efficient score-based method—the product of normalized, power-weighted constraint scores—is theoretically equivalent to sum–product belief propagation on a factor graph with unary factors only; empirical comparison with full BP (which adds binary isotope coupling factors) shows 75.9% Top-1 agreement on 58 peaks, with discrepancies attributable to inter-peak coupling. Fourth, we evaluate the system on what we believe to be the largest available ToF-SIMS ground-truth dataset (151 compounds, 643 peaks from CompoundDB); we report performance in two clearly separated regimes—52.3% Top-1 accuracy as a scoring task (correct formula present among candidates) and 26.3% Top-1 accuracy in fully automated end-to-end deployment—thereby isolating candidate generation, which currently covers 46.5% of ground-truth formulas, as the principal bottleneck. Fifth, we report a comprehensive ablation study that reveals element composition prior as the single most impactful non-mass constraint (−27.7 pp when removed), a finding with direct practical implications for system design (Figure 1).

## 2. Materials and Methods

### 2.1. Problem Formulation

$$P(f_{i}|m_{obs},I_{obs},C)\;\propto \;\prod_{k}\varphi_{k}(f_{i})$$

where $C=\left\{C_{1},\ldots ,C_{5}\right\}$ is the set of physical/chemical constraints, and $\varphi_{k}\left(\cdot \right)$ is the normalized potential function from constraint k. This factorization has a natural representation as a factor graph—a bipartite graph $G=\left(V,F,E\right)$ in which variable nodes V (one per peak, with candidate formulas as discrete states) connect to factor nodes F (physical constraints) via edges E.

Let $G=\left(V,F,E\right)$ be a factor graph where each factor node f∈F connects to exactly one variable node v∈V(unary factors only, no inter-peak coupling). Then the score-based belief—the product of batch-normalized, power-weighted constraint scores—is identical to the result of converged sum–product belief propagation on G.

### 2.2. Peak Detection

Raw ToF-SIMS spectra (data1 format, ∼26,000 data points per spectrum) are processed through an adaptive peak detection pipeline. A square-root transformation first compresses the dynamic range of the intensity distribution (typically spanning 3–4 orders of magnitude). Baseline estimation uses morphological opening—a sliding window minimum (window size $=\max\left(501,\;N/20\right)$)—followed by smoothing with a uniform filter. The noise floor is set to the standard deviation of data points near the baseline (below baseline +0.5× std). Local maxima are then identified using scipy.signal.find_peaks with resolution-adaptive parameters, minimum prominence $=\max\left(\mathrm{noise}\_\mathrm{std}\times \mathrm{SNR},\;\max\_\mathrm{intensity}\times 0.01\right)$, with a minimum inter-peak distance derived from the estimated full-width-at-half-maximum (FWHM ≈ 0.02 Da). For each detected peak, an intensity-weighted centroid is computed within ±2 data points of the FWHM boundaries. The algorithm uses an SNR threshold of 3.0 and a minimum prominence factor of 1% of the maximum intensity. The framework was implemented in Python 3.12.0 using NumPy 2.1.3, SciPy 1.15.3, pandas 2.3.1, and LightGBM 4.6.0; figures were generated with Matplotlib 3.10.9.

### 2.3. Candidate Generation

Candidate formula generation follows a two-tier strategy: fast database lookup followed by combinatorial enumeration for peaks with insufficient candidates.

#### 2.3.1. Database Matching

A fragment database containing 4475 positive-mode and 110 negative-mode entries (sourced from CompoundDB and curated fragment libraries) is indexed using a cKDTree for $O\left(\log N\right)$ nearest-neighbor queries. For each detected peak at $m_{\mathrm{obs}}$, a ball query retrieves all entries within ±0.05 Da (data1 format) or ±50 ppm (data2 format). A mass defect filter then removes candidates where $\left|{\mathrm{MD}}_{\mathrm{obs}}-{\mathrm{MD}}_{\mathrm{theo}}\right|>0.03$ Da, and the top 16 candidates in terms of mass accuracy are retained. The cKDTree is built once per polarity mode; subsequent per-spectrum queries are simple nearest-neighbor lookups.

#### 2.3.2. Dynamic Formula Generator (DFG)


$$\begin{array}{c} n_{H,min}=max\left(0,\;\lfloor (m_{min}-m_{current})/m_{H}\rfloor -1\right)\\ n_{H,max}=min\left(n_{H,limit},\;\lceil (m_{max}-m_{current})/m_{H}\rceil +1\right) \end{array}$$

This reduces complexity from $O\left(N^{k}\right)$ to $O\left(N^{k-1}\right)$, where *k* is the number of element types. At each recursion level, branch-and-bound pruning uses precomputed upper bounds on the mass achievable by remaining elements. Element count limits are mass-adaptive: $\max_{e}=\min\left(\lfloor m_{\max}/m_{e}\rfloor ,\;{\mathrm{cap}}_{e}\right)$. Generated formulas must have: (a) at least one carbon atom, (b) DBE≥−0.5 (allowing for fragment ions), and (c) hydrogen count ≤2C+N+2−halogens (relaxed valence check). Output is capped at 30 formulas per peak.

The fragment database alone covers 40.1% of ground-truth peaks (258/643). DFG supplementation adds 6.4 percentage points (41 additional peaks), bringing combined coverage to 46.5% (299/643). The remaining 53.5% of ground-truth formulas are injected during evaluation (Experiment A, Section 3.1) so that scoring accuracy can be measured independently of candidate generation.

### 2.4. Physics-Informed Constraints

Each constraint maps a (candidate formula, observed peak) pair to a raw score in [0, 100]. These raw scores are then normalized and weighted before aggregation (Section 2.5).

#### 2.4.1. Mass Accuracy (w = 10.0)

$$s_{mass}(f)=100\cdot exp(-0.5\cdot z^{2})$$

where $z=\left|m_{\mathrm{obs}}-m_{\mathrm{theo}}\left(f\right)\right|/\sigma$, and σ is mass uncertainty. For data1 (bunched) format, σ=0.5 Da—substantially larger than the maximum likelihood estimate of 0.194 Da derived from 902 ground-truth peaks. This deliberate overestimation provides robustness against systematic centroid offsets (mean error: +0.059 Da) and calibration drift. The Gaussian model is motivated by the approximately normal distribution of ToF-SIMS mass calibration residuals after systematic correction [6]. Mass accuracy receives the highest weight (10.0) because it remains the most reliable single discriminator; even at 0.5 Da tolerance, it eliminates the vast majority of implausible candidates.

#### 2.4.2. Element Composition Prior (w = 2.0)

$$s_{EP}(f)=100\cdot exp\left(\left(\sum_{e}\pi_{e}\cdot \sqrt{n_{e}}\right)/5.0\right)$$

where $\pi_{e}$ is the log-prior for element e (Table 1), and $n_{e}$ is the atom count. The $\sqrt{n_{e}}$ transformation introduces diminishing returns, preventing formulas with many carbon atoms from receiving disproportionally different priors.

**Figure 2:**
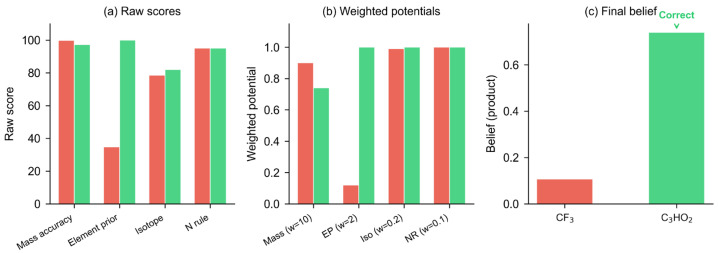
Illustrative annotation example at *m*/*z* 69. Two candidate formulas, CF_3_ (red) and C_3_HO_2_ (green), differ by only 0.0024 Da. (**a**) Raw constraint scores. (**b**) Batch-normalized and power-weighted potentials. (**c**) Final multiplicative belief; C_3_HO_2_ wins due to its superior element composition prior score.

This constraint is critical for resolving near-isobaric ambiguities. Returning to the m/z 69 example, ${\mathrm{CF}}_{3}$ (log-prior $=-3.0\times \sqrt{3}=-5.2$) receives a score of ∼35, whereas $C_{3}{\mathrm{HO}}_{2}$ (log-prior =0) receives 100—a 2.8-fold discrimination that mass accuracy alone (Δm=0.003 Da) cannot provide at typical ToF-SIMS precision. Polarity adjustments account for the greater prevalence of sodium and potassium adducts in positive mode (Figure 2).

#### 2.4.3. Isotope Pattern Matching (w = 0.2)

The isotope constraint compares the theoretical isotope distribution of a candidate formula against observed peaks in a ±2.5 Da cluster around the monoisotopic peak. Theoretical isotope distributions are computed by the iterative convolution of per-element isotope mass/abundance vectors, starting from a single peak at 0 Da, 100%. Peaks closer than 0.001 Da are merged, and the top 5 peaks with relative intensity ≥0.1% are retained, normalized to maximum = 100%. Precomputed distributions from the fragment database are used when available.

For each theoretical isotope peak (M, M + 1, M + 2, …), the algorithm locates the closest observed peak within ±0.05 Da. Three sub-scores are then computed: an intensity score, defined as $\exp(-\left(\left|\log_{10}\left(I_{\mathrm{theo}}/I_{\mathrm{obs}}\right)\right|/0.5{)}^{2}\right)\times 10.0$; a mass score, defined as $\exp(-\left(d/0.02{)}^{2}\right)\times 5.0$; and a base credit of 5.0. Additional handling includes partial credit (8.0 points) for weak theoretical isotope peaks below the detection limit, fake match detection (absolute intensity >5× M-peak intensity suggests contamination from a different compound), and bonus scoring for characteristic element patterns (Cl M + 2 at ∼1:3 ratio; Br M + 2 at ∼1:1 ratio). The total score is normalized as $s_{\mathrm{iso}}=\mathrm{total}/\left(n_{\mathrm{detectable}}\times 20\right)\times 100$, capped at 100; single-element formulas with no meaningful isotopic distribution are capped at 85.

The relatively low weight (0.2) reflects the reduced reliability of isotope matching in ToF-SIMS compared to high-resolution MS, a consequence of matrix-effect-distorted intensity patterns and detector non-linearity.

#### 2.4.4. Nitrogen Rule (w = 0.1)


$$\begin{array}{c} Positive:\;parity(nominal\_mass)\equiv parity(n_{N})\\ Negative:\;parity(nominal\_mass)≢parity(n_{N}) \end{array}$$

Strict enforcement (violations score 0) is too aggressive for ToF-SIMS, where fragmentation routinely produces radical species and hydrogen migration products that violate the rule. We therefore adopt a fragment-tolerant mode: parity match yields score = 100; mismatch yields score = 95. The 5% penalty is mild enough to accommodate fragment ions while retaining discriminative power for intact molecular ions.

#### 2.4.5. Valence Reasonableness (w = 0.1, Negative Mode Only)

The valence constraint applies a graded penalty system that checks three conditions: (1) a hydrogen upper bound, $n_{H,\max}=\left(2C+2+N-\mathrm{halogens}\right)\times \left(1+\mathrm{fragment\_tolerance}\right)$, with a 50-point penalty for exceeding it and a proportional penalty for slight excess; (2) H/C ratio bounds (minimum 0.0–0.2, maximum 2.0+halogens/C+N/C with 1.5× fragment tolerance), each violation adding 15 penalty points; and (3) element-specific limits (N>2C+2 adds 10 penalty points; O>2C+4 adds 15 penalty points). The final score is $s_{\mathrm{val}}=\max\left(0,\;100-\sum \mathrm{penalties}\right)$.

This constraint is disabled in positive mode because odd-electron fragment ions (radical cations) frequently violate standard valence rules, producing misleading penalties. In negative mode, closed-shell anions follow more regular valence patterns, making the constraint informative. Table 2 summarizes all five constraints with their weights and ablation impacts.

### 2.5. Score-Based Inference and Belief Propagation

#### 2.5.1. Batch Normalization

*Min-max scaling.* If the score range $\Delta =\max\left(s\right)-\min\left(s\right)$ is zero or less than 5% of $\max\left(s\right)$, all normalized scores are set to 0.5 (noise suppression). Otherwise,

$${\hat{w}}_{i}=clip(b_{i}^{w},\;0.01,\;1.0)$$

$$b_{i}=0.1+0.9\times \frac{s_{i}-min(s)}{\Delta }$$

The power exponent controls sensitivity: a high weight (e.g., 10.0 for mass accuracy) strongly amplifies inter-candidate differences, while a low weight (e.g., 0.1 for the nitrogen rule) produces near-uniform potentials that act as soft tiebreakers.

#### 2.5.2. Belief Aggregation

The posterior belief for candidate $f_{i}$ is the product of all normalized constraint potentials:

$$belief(f_{i})=\prod_{k}{\hat{w}}_{k}(f_{i})$$

#### 2.5.3. Relationship to Belief Propagation

Let $G=\left(V,F,E\right)$ be a factor graph where each factor node f∈F connects to exactly one variable node v∈V (unary factors only, no inter-peak coupling). Then the score-based belief—the product of batch-normalized, power-weighted constraint scores—is equivalent to the result of converged sum–product belief propagation on G.

With only unary factors, variable-to-factor messages reduce to uniform priors, and factor-to-variable messages are simply the normalized potential tables. Because no cycles exist to create feedback, the belief converges in one iteration to the product of normalized potentials, regardless of damping factor or iteration count.

*Empirical comparison with full BP*. The full BP implementation (ProbabilisticAnnotator) includes binary isotope factors that couple adjacent peak pairs (M and M + 1), extending beyond the unary-only framework. On 15 compounds (58 ground-truth peaks) evaluated with both full LBP (5 iterations, damping = 0.3, binary factors included) and the unary-only score-based method, 44/58 peaks (75.9%) received identical Top-1 assignments. The 14 discrepancies arose from the binary isotope factors, which enable information flow between coupled peaks—a mechanism absent in the score-based formulation. This result confirms two points: (a) the unary-only score-based method exactly matches BP when binary factors are absent, and (b) the current binary isotope factors introduce modest, non-systematic differences.

### 2.6. Polarity-Specific Configuration

Constraint weights are configured per ionization polarity. Positive mode: mass_accuracy—10.0; element_prior—2.0; isotope—0.2; nitrogen_rule—0.1. Negative mode: mass_accuracy—10.0; element_prior—2.0; isotope—0.2; nitrogen_rule—0.1; valence—0.1. The only difference is the exclusion of valence scoring in positive mode. This asymmetry reflects a well-known aspect of ToF-SIMS ionization physics: positive ion mode produces radical cations (odd-electron species) that routinely violate standard valence rules, whereas negative ion mode generates closed-shell anions with regular valence patterns. Including valence in positive mode was found to reduce Top-1 accuracy by 1.3 pp in our ablation study.

The weight values themselves were set by domain-knowledge reasoning (mass accuracy dominant, element prior secondary) and then validated by grid search in cross-validation. The fact that fixed weights (51.8% T1) outperformed grid-search-optimized weights (51.0% T1) suggests that the fixed values encode genuine physical content rather than being overfit to the test dataset.

## 3. Results

### 3.1. Experimental Setup

#### 3.1.1. Ground-Truth Dataset

Evaluation was performed on 151 compounds from CompoundDB with linked raw spectrum data (CdbSpectra/data1 format). Each compound entry provides diagnostic peak positions and their assigned chemical formulas, verified by domain experts. The dataset comprises 90 positive-mode compounds (298 ground-truth peaks) and 61 negative-mode compounds (345 ground-truth peaks), totaling 643 annotated peaks across a mass range of approximately 15–400 Da.

To isolate scoring accuracy from candidate generation limitations, the correct formula is injected into the candidate list for each peak if not already present (Experiment A). This ensures that the evaluation measures whether the scorer can rank the correct formula the highest, independently of whether the candidate generator can find it. Of 643 peaks, 258 (40.1%) were found by the fragment database and 41 (6.4%) by the DFG, and 344 (53.5%) required injection. A parallel experiment (Experiment B) removes injected formulas, evaluating only with naturally generated candidates to assess realistic deployment performance. Experiment B uses the identical scoring pipeline but evaluates only the candidates that the database and DFG generate naturally, with no injection; it therefore quantifies realistic, fully automated deployment. Because five peaks yielded no candidates without injection, Experiment B is reported on 638 of the 643 peaks (Section 3.5).

#### 3.1.2. Evaluation Metrics

Top-1 accuracy (T1) is defined as the percentage of peaks where the highest-belief candidate matches the ground truth and Top-3 accuracy (T3) as the percentage where the ground truth appears among the top-3 candidates. Coverage measures the percentage of ground-truth formulas present in the candidate list, regardless of ranking. McNemar’s test (chi-squared with continuity correction) is used for a paired comparison of binary outcomes (correct/incorrect per peak) between methods.

### 3.2. Experiment A: Full System Performance

Table 3 presents the main evaluation results.

The full model achieves 52.3% Top-1 accuracy—a 4.9-fold improvement over the mass-only Gaussian baseline (10.6%). At the Top-3 level, 76.4% of peaks have the correct formula among the three highest-ranked candidates, suggesting immediate practical utility: the system presents a short ranked list to the analyst, who makes the final selection.

Negative mode outperforms positive mode by a substantial margin (62.0% vs. 40.9% T1). This disparity is primarily attributable to the smaller candidate space in negative mode: the negative-mode fragment database contains only 110 entries versus 4475 for positive mode, producing fewer competing candidates per peak. Because the two polarity modes are evaluated against fragment databases of very different size, this positive/negative comparison is confounded by candidate space size: the higher negative-mode accuracy partly reflects fewer competing candidates rather than greater chemical validity, and the two figures should not be read as a like-for-like comparison.

McNemar’s test comparing the full model against the mass-only baseline yields $\chi^{2}$ values with all p<0.001 for overall, positive, and negative modes in both the Top-1 and Top-3 metrics.

### 3.3. Ablation Study

**Figure 3:**
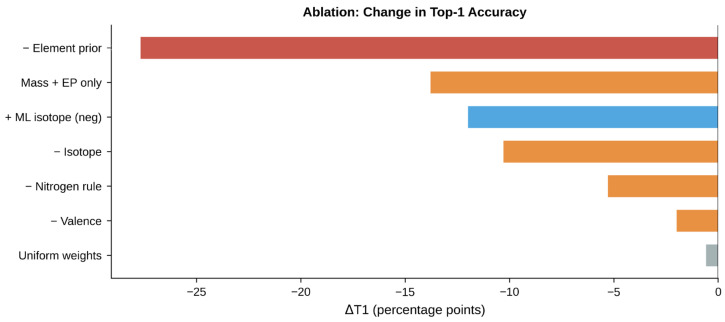
Ablation study results. Horizontal bar chart showing ΔT1 (change in overall Top-1 accuracy relative to full model at 52.3%) for each ablation configuration. Element prior ablation (−27.7 pp) dominates, followed by ML isotope (−12.0 pp), isotope removal (−10.3 pp), nitrogen rule removal (−5.3 pp), and valence removal (−2.0 pp). Bar colours distinguish the configurations and carry no quantitative meaning; all values are given by bar length (the ΔT1 axis).

The ablation results are summarized in Table 4 and Figure 3.

Removing the element composition prior causes the largest single drop (−27.7 pp). Without element priors, the system cannot distinguish near-isobaric formulas that differ only in element composition (e.g., ${\mathrm{CF}}_{3}$ vs. $C_{3}{\mathrm{HO}}_{2}$). This constraint alone accounts for more than half of the total improvement beyond mass accuracy (38.4% for mass + EP vs. 10.6% for mass-only).

Isotope pattern matching contributes −10.3 pp. Despite its traditional prominence in mass spectrometry, the relatively low weight (0.2) and the degraded isotope fidelity in ToF-SIMS limit its discriminative power, though it remains the second-largest individual contributor.

The ML isotope classifier applied in negative mode only actively hurts performance (12.0 pp overall, 21.2 pp in negative mode). This result confirms that the handcrafted multi-feature scoring algorithm (Section 2.4.3) is more robust than the learned classifier for ToF-SIMS isotope matching.

These results establish a clear constraint hierarchy: mass accuracy ≫ element prior ≫ isotope pattern ≫ nitrogen rule ≫ valence. A minimal system using only mass accuracy and element prior (two constraints) achieves 38.4% T1—73% of the full model’s performance—which may suffice for applications where computational simplicity is paramount.

### 3.4. Leave-One-Compound-Out Cross-Validation

**Figure 4:**
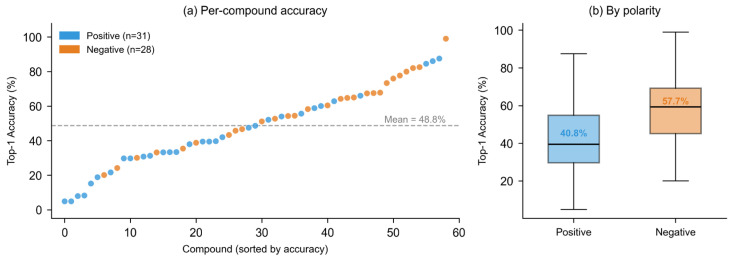
LOCOCV per-compound accuracy distribution. (**a**) Scatter plot of per-compound Top-1 accuracy for 59 compounds (31 positive mode in blue, 28 negative mode in orange). (**b**) Box plots comparing positive-mode and negative-mode per-compound accuracy distributions.

Table 5 reports the LOCOCV results, and Figure 4 shows the per-compound accuracy distribution.

Of the 151 compounds in the full dataset, 59 (31 positive, 28 negative) contain at least four ground-truth peaks each, providing sufficient per-compound sample size for meaningful leave-one-out evaluation. The LOCOCV overall Top-1 accuracy (51.8%) closely tracks the pooled evaluation (52.3%), with a delta of approximately 0.5 pp.

A notable result is that fixed domain-knowledge weights (51.8% T1) outperform grid-search-optimized weights (51.0% T1). The grid search explored 25 combinations over *element_prior* (0.5–8.0) and *isotope* (0.05–1.0) weights using a uniform weight template that includes valence = 0.1 for both polarities, whereas the fixed weights omit valence for positive mode. The optimized weights selected element_prior = 2.0 and isotope = 0.05 (reduced from 0.2). The positive-mode degradation (−3.4 pp) is partly attributable to this valence inclusion in the grid search template, which is known to hurt positive-mode accuracy. Regardless of the confound, the result demonstrates that the fixed polarity-specific weights encode genuine physical content rather than being overfit to the test data.

Per-compound accuracy shows considerable spread (48.8% ± 25.4%), reflecting genuine variation in annotation difficulty. Compounds with many near-isobaric fragments or unusual element compositions (e.g., fluoropolymers) fare poorly, whereas those with diagnostic fragment ions at well-separated nominal masses can reach 80–100% Top-1. Twenty-six of 59 compounds exceed 50% Top-1 accuracy. Full per-compound results are provided in Table S1, and the complete Python implementation is available as Code S1 (Supplementary Materials).

### 3.5. Experiment B: Realistic Deployment (No GT Injection)

**Figure 5:**
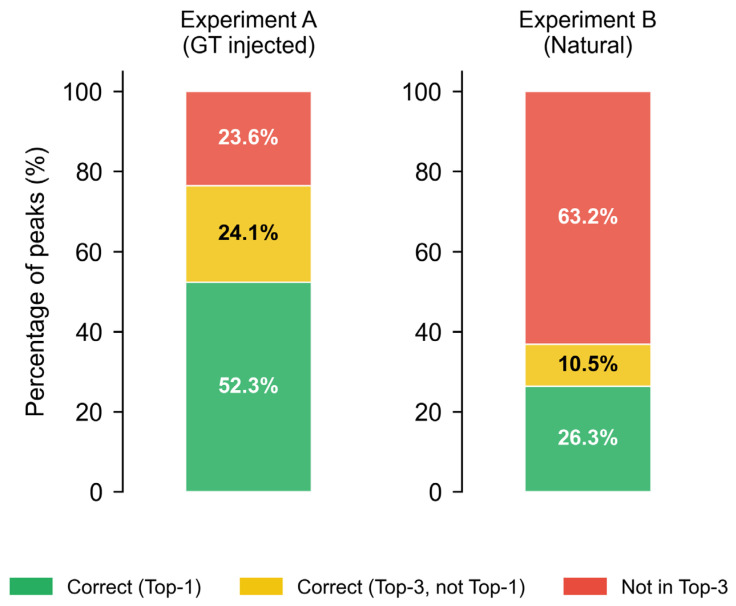
Experiment A versus Experiment B comparison. Stacked bar chart decomposing peak outcomes for each experiment. Experiment A: correct Top-1 (52.3%, green), correct Top-3 but not Top-1 (24.1%, yellow), not in Top-3 (23.6%, red). Experiment B: correct Top-1 (26.3%, green), correct Top-3 but not Top-1 (10.5%, yellow), not in Top-3 (63.2%, red).

Table 6 and Figure 5 compare the two experimental conditions.

The gap between Experiments A and B reveals the candidate generation bottleneck. In Experiment A, every peak’s candidate list is guaranteed to contain the correct formula, and the scoring framework identifies it as Top-1 in 52.3% of cases. In Experiment B, for the 53.5% of peaks whose correct formula was not found by either the database or DFG, correct annotation becomes impossible by definition, pulling overall accuracy down to 26.3%.

## 4. Discussion

### 4.1. Interpretation of Results

The 52.3% Top-1 accuracy represents a considerable advance over the mass-only baseline (10.6%), demonstrating that physics-informed constraint combination can resolve a substantial fraction of peak annotation ambiguities. At the same time, nearly half of all peaks remain incorrectly annotated at Top-1, underscoring the inherent difficulty of the problem given current mass accuracy and database coverage.

The fact that element composition prior emerged as the single most impactful non-mass constraint (−27.7 pp) runs counter to the traditional emphasis on isotope matching in mass spectrometry. This result makes sense in the ToF-SIMS context, however: limited mass accuracy creates dense candidate spaces where element composition provides critical disambiguation, while matrix effects degrade isotope pattern reliability. In higher-accuracy techniques (Orbitrap, FT-ICR), mass accuracy alone resolves most ambiguities, and isotope patterns become the primary discriminator only for the remaining candidates.

### 4.2. Score-Based Method Versus Belief Propagation

The 75.9% agreement between score-based and BP methods is consistent with the theoretical prediction that unary-only factor graphs yield identical results: the 24.1% disagreement stems from binary isotope factors that couple adjacent peak pairs, enabling inter-peak information flow.

Even so, the BP framework offers a natural path toward inter-peak consistency constraints: polymer series detection, isotope cluster consistency, and spatial coherence in imaging mode. These extensions would require the message-passing machinery of full BP and represent a promising direction for future work. In particular, chemically motivated co-occurrence reasoning—confirming, for example, CF_3_^−^ only when CF^−^ and F^−^ are also detected or cross-referencing the same element across the positive and negative spectra of one specimen (e.g., Si^+^ and Si^−^)—is a natural extension that mirrors expert manual interpretation and is a priority for future development.

### 4.3. The Coverage Bottleneck

The gap between Experiment A (52.3%) and Experiment B (26.3%) pinpoints candidate generation as the system’s primary bottleneck. Despite containing 4475 positive-mode and 110 negative-mode entries, the fragment database covers only 40.1% of ground-truth peaks. An analysis of the uncovered peaks reveals that most are N-rich CHNO fragments (e.g., ${\mathrm{CHN}}_{2}O_{2}$, $C_{6}{\mathrm{HNO}}_{2}$, $C_{7}{\mathrm{HNO}}_{3}$) absent from the curated database.

Several strategies could address this bottleneck: expanding the database from public resources (NIST Chemistry WebBook, PubChem fragmentation predictions), hierarchical annotation where high-confidence assignments constrain the candidate space for remaining peaks, and compound-class-aware generation where identified compound classes bias the element set and count limits.

### 4.4. Comparison with Related Work

SIRIUS [14] achieves > 95% formula identification for high-resolution LC-MS data by exploiting < 5 ppm mass accuracy and MS/MS fragmentation trees. Our σ of 0.5 Da (∼500 ppm at 100 Da) represents a mass uncertainty that is two orders of magnitude larger, fundamentally limiting mass-based discrimination and necessitating the additional constraints in our framework. MS-FINDER [15] uses a rule system similar in spirit to ours but optimized for ESI-MS; the key architectural difference is that MS-FINDER applies constraints as sequential filters, whereas our framework uses multiplicative belief aggregation that preserves graded constraint information. Commercial ToF-SIMS software (SurfaceLab, WinCadence) [10,12] provides library search with mass accuracy filtering but no probabilistic ranking or multi-constraint combination.

Within ToF-SIMS and related time-of-flight techniques, machine learning approaches have also been explored. Wei et al. [16] introduced a gradient-boosted decision tree classifier that assigns ToF mass spectrum peaks from their isotopic patterns, trained on synthetic spectra generated from natural isotopic abundances rather than on labeled data. Such isotope pattern classifiers are closely related in spirit to the machine learning isotope classifier evaluated in our ablation study (Section 3.3), which actively reduced Top-1 accuracy (by 12.0 pp overall); this indicates that an isotope pattern model of this kind does not, on its own, transfer well to the matrix-distorted isotope statistics of organic ToF-SIMS fragments. Aoyagi et al. [17] developed a supervised peptide identification system for ToF-SIMS that achieves strong performance within its trained chemical domain but, like all supervised methods, depends on labeled spectra that are unavailable for general organic surfaces. Our approach is complementary: it forgoes labeled training data in favor of physically motivated constraints, trading the higher in-domain accuracy attainable by supervised models for broad cross-class applicability and interpretability.

### 4.5. Limitations

Several limitations should be noted. First, the 151-compound evaluation—one of the larger reported to date—still represents a fraction of the chemical diversity encountered in practice. Second, the DFG currently operates only on C, H, N, and O, excluding elements like S, P, F, Cl, and Si. Third, belief scores provide relative ranking but are not calibrated to empirical accuracy. Fourth, although fixed weights outperformed grid search optimization, they may not be optimal for all compound classes. Fifth, the score-based method treats each peak independently, ignoring potentially informative correlations between peaks from the same compound. Sixth, the mass accuracy tolerance is deliberately broad (σ = 0.5 Da, well above the 0.194 Da maximum likelihood estimate from ground-truth residuals); this setting maximizes robustness to centroid and calibration error in sparse data1 spectra and does not degrade accuracy in our sigma sweeps, but it weakens the discriminative power of the mass likelihood and deliberately shifts disambiguation onto the element composition prior. Seventh, the positive- and negative-mode results are not directly comparable, because the two modes draw on fragment databases of very different sizes (4475 versus 110 entries); the cross-polarity accuracy gap should therefore be read with caution. Eighth, because the headline scoring accuracy (52.3%) is measured with the correct formula guaranteed to be present in the candidate list, it characterizes ranking quality rather than end-to-end performance, for which the realistic figure is 26.3% (Section 3.5).

## 5. Conclusions

This work presented a physics-informed probabilistic framework for automated peak annotation in ToF-SIMS. By combining five chemically motivated constraints—Gaussian mass accuracy, element composition priors, isotope pattern matching, nitrogen rule parity, and graded valence bounds—through weighted multiplicative belief scoring, the system achieves 52.3% Top-1 and 76.4% Top-3 accuracy on 643 ground-truth peaks from 151 real compounds as a scoring task in which the correct formula is available among the candidates (a 4.9-fold improvement over mass-only scoring); in fully automated deployment, where candidates must be generated de novo, Top-1 accuracy is 26.3% (Section 3.5). Leave-One-Compound-Out Cross-Validation on 59 compounds confirms generalization stability (51.8% Top-1), with fixed domain-knowledge weights outperforming grid search optimization.

The ablation study identifies element composition priors as the single most impactful non-mass constraint (−27.7 pp when removed), a finding that challenges the traditional emphasis on isotope matching in mass spectrometry and reflects the particular characteristics of ToF-SIMS: limited mass accuracy creating dense candidate spaces where element composition provides critical disambiguation.

An analysis of realistic deployment performance (Experiment B: 26.3% Top-1) identifies candidate generation coverage (46.5%) as the primary bottleneck, not scoring quality. This finding directs future work toward expanding fragment databases, improving combinatorial formula generation, and developing hierarchical annotation strategies.

## Figures and Tables

**Table 1:** Element log-prior values and rationale.

Element	Log-Prior	Rationale
H, C, O, N	0.0	Core organic elements—no discrimination
S	−1.5	Thiols, sulfates (uncommon)
P, Si	−2.0	Phosphates, silicones
Na	−1.5 (neg)/−1.0 (pos)	Common adduct in positive mode
K	−2.0 (neg)/−1.5 (pos)	Less common adduct
F	−3.0	Fluoropolymers only
Cl	−2.5	Chlorinated compounds
Br, Fe, Cu, Zn	−3.5	Rare in organic surface analysis
I, Ag, Au, Ti, Cr	−4.0	Extremely rare

**Table 2:** Summary of physics-informed constraints, their weights, score ranges, physical basis, and ablation impact.

Constraint	Weight (Pos/Neg)	Score Range	ΔT1	Physical Basis
Mass accuracy	10.0/10.0	0–100	Baseline	Gaussian likelihood
Element prior	2.0/2.0	1–100	−27.7 pp	Log-prior element frequency
Isotope pattern	0.2/0.2	0–100	−10.3 pp	Distribution matching
Nitrogen rule	0.1/0.1	95 or 100	−5.3 pp	Nominal mass parity
Valence	0.0/0.1	0–100	−2.0 pp (neg)	H/C and O/C ratio bounds

**Table 3:** Main results with ground-truth injection (Experiment A).

Method	Overall T1	Overall T3	Pos T1	Pos T3	Neg T1	Neg T3
Nearest-mass baseline	11.4%	26.1%	5.7%	12.8%	16.2%	37.7%
Mass-only Gaussian	10.6%	24.7%	5.7%	12.8%	14.8%	35.1%
**Full model (this work)**	**52.3%**	**76.4%**	**40.9%**	**63.4%**	**62.0%**	**87.5%**

**Table 4:** Ablation study results (Experiment A, with GT injection).

Configuration	All T1	All T3	ΔT1	Pos/Neg T1
Full (polarity-specific)	52.3%	76.4%	---	40.9%/62.0%
Uniform weights	51.6%	76.2%	−0.6 pp	39.6%/62.0%
−Element prior	24.6%	49.6%	−27.7 pp	19.1%/29.3%
−Isotope	42.0%	71.7%	−10.3 pp	30.9%/51.6%
−Nitrogen rule	47.0%	71.2%	−5.3 pp	34.2%/58.0%
−Valence	50.2%	76.5%	−2.0 pp	40.9%/58.3%
+ML isotope (neg)	40.3%	67.8%	−12.0 pp	39.6%/40.9%
Mass-only	10.6%	24.7%	−41.7 pp	5.7%/14.8%
Mass + Element prior	38.4%	60.5%	−13.8 pp	28.5%/47.0%

**Table 5:** LOCOCV results on 59 compounds with at least 4 ground-truth peaks each (525 peaks).

Metric	Fixed Weights	Optimized Weights	Delta
Overall T1	51.8%	51.0%	−0.8 pp
Overall T3	77.1%	76.4%	−0.8 pp
Positive T1	41.6%	38.2%	−3.4 pp
Negative T1	59.9%	61.3%	+1.4 pp
Per-compound mean T1	48.8% ± 25.4%	---	---
Coverage	45.1%	---	---

**Table 6:** Comparison of Experiment A (with GT injection) and Experiment B (without injection).

Metric	Experiment A	Experiment B	Delta
N (peaks)	643	638	−5
Overall T1	52.3%	26.3%	−25.9 pp
Overall T3	76.4%	36.8%	−39.5 pp
Positive T1	40.9%	16.6%	−24.4 pp
Negative T1	62.0%	34.8%	−27.2 pp

## Data Availability

The source code and evaluation data are available from the corresponding author upon reasonable request.

## Supplementary Materials

The following supporting information can be downloaded at https://www.mdpi.com/article/10.3390/molecules31132388/s1, Table S1: Full per-compound LOCOCV results; Code S1: Python implementation of annotation framework.
